# Supplementary material for: A size and space structured model of tumor growth describes a key role for protumor immune cells in breaking equilibrium states in tumorigenesis
Source: PLoS One. 2021 Nov 22;16(11):e0259291. doi: 10.1371/journal.pone.0259291 (PMC8608488; doi:10.1371/journal.pone.0259291)
Supplement: Attachment — Submitted filename: Atsou et al-Rebuttal letter-Responses to reviewers.pdf [file pone.0259291.s001.pdf]

A size and space structured model of tumor growth describes a key role for protumor immune cells in breaking equilibrium states in tumorigenesis

Kevin Atsou, Fabienne Anjuère, Véronique M. Braud, and Thierry Goudon

Transfer from Plos Computational Biology to Plos One

Point-by-point responses to reviewers of Plos Computational Biology

Reviewer #1: The manuscript by Atsou et al. does not fall within the PLoS Computational Biology scientific scope:
"PLOS Computational Biology features works of exceptional significance that further our understanding of living systems at all scales—from molecules and cells, to patient populations and ecosystems—through the application of computational methods" for the following reasons:

1. has a limited link to biological reality and thus will not bring any new insights into tumor growth and tumor-immune system interactions

We think this assessment is unfair. In the modeling approach, one needs to make simplifying assumptions, that certainly departs from the reality, but which allow us to bring out the main features of the physico-biological mechanisms. Addressing all phenomena of the immune system tumor interaction in a set of equations is completely unrealistic.
Besides, our model reproduces equilibrium and escape phases that are observed in cancer patients; thus it can provide useful informations on the features of these phases and it is biologically relevant.

2. several distinct immune cells are collapsed together (T cells, NK cells, neutrophils, macrophages) even if they have different developmental properties and diverse mechanism of interactions with tumor cells — which may lead to misleading results

As said in #1, this is intrinsic to the modeling approach. We definitely do not believe that it could be realistic, nor informative, to write an equation for each type of cell.

3. tumors are known to be highly heterogeneous, and yet the authors assume constant microenvironmental conditions

Sure. However, it is still reasonable to start, especially to model the earliest stages of tumor growth, with this simplifying framework. Dealing with more intricate microenvironmental conditions leads to the difficulty of fixing further parameters of the model. Is the reviewer able to provide them ? or does it mean that he renounces to any modeling effort ?

4. how the immune cell displacement can be infinitely large? immune cell movement is on much smaller scale than cell size

This is definitely a wrong statement: immune cells move from the lymph nodes to the location of the tumor, which can be a displacement of several cm., far larger than the size of tumor cells (with radius about 10 micrometers). This has been clarified in the revised version.

5. model parameters have not values and no units in Table 1 showing again that this is more a mathematical exercise than a model with biological meaning; model parameters values should

be based on biological measurements to have impact on real life phenomena; the same, no units are reported in all figures so there is no indication whether they have any biological relevance

Many parameters can be found in the literature and such a Table is provided in our previous works. However, there still remain unknown values. It is not obvious to design experimental procedures to determine them, and they need to be found indirectly from experimental data. Such data are not always well adapted (for instance they rarely give a time-evolution of the tumor growth because they are acquired by sacrificing mice). We do not believe that a fitting procedure, simply based on commercial softwares, can provide a relevant set of parameters for our purposes. We also point out that such procedures are quite well developed as far as one deals with ordinary differential equations, but are less advanced for partial differential equations. Setting up methods to calibrate the parameters for experimental data would be a very valuable task, that deserves a paper (at least!) in itself, and this is far beyond the modeling purposes of the present paper.

6. no discussion on whether the obtained results/figures are achieved for biologically relevant conditions/parameter values, which again confirms there is a little attention given to biological reality, and the presented work is simply an academic exercise

What is remarkable here is that the model is already able to reproduce qualitative features of biological observations, without any built-in arguments. Numerical results in this paper have only the goal to illustrate the qualitative features of the solutions and the role of the parameters, in line with the biological interpretation. We indeed do not claim in this work that the parameter values are biologically relevant, since we think a detailed parameter calibration would obscure the purposes of the paper, which is oriented towards the modeling issues. Moreover, identifying and calibrating these values lead to further challenging questions that require the development of specific methods (optimization, statistical analysis…) and deserve a publication in themselves.

7. all stability, equilibrium and theorems will not hold in reality due to unrealistic model simplifications. Overall, this is purely academic exercise and will fit better into mathematical journal.

Any modeling approach is based on assumptions, that are certainly not fully satisfied. This does not mean that a theorem on such a simplified description has no value. It provides a basis for the explanation of the observed phenomena, at least in certain regimes. Thus, it opens doors to discuss the governing mechanisms of these phenomena.
This comment reflects a lack of scientific openness. It is really surprising that a reviewer for Plos Comput. Biol. is so negative about what is just the basis of the modeling approach.

Reviewer #2: The authors present and analyse a mathematical model, formulated as a system of PDEs, to investigate tumour-immune cell interactions. Of interest is understanding how the composition of the immune cell population changes as the tumor develops and, in particular, how a shift towards a pro-tumor phenotype facilitates tumor growth and expansion. Indeed, the novelty of this model, in comparison to the authors' previous work,sis the inclusion of a pro-tumour immune population. The authors use their model to investigate the efficacy of treatments that boost the antitumor immune response and/or reduce the protumor immune response. The results appear to be technically correct but I have not checked them carefully. As

such, the research presented is highly topical, likely to be of wide interest and publishable (after some revisions).
My main and major concern with the paper is its suitability for publication in PLOS Computational Biology. The model is complex (size- and space-structured model, with nonlocal terms) and its description is difficult to follow. The numerical method was developed in an earlier publication of the authors (no details are provided in the current manuscript).

This is correct: there is no new insight about the numerical methods here.

The numerical results are rather limited in their extent. In particular, an extensive parameter sensitivity analysis is not provided, and nor is the model validated against experimental data. It would have been informative, for example, to present a bifurcation diagram showing how the number, nature and stability of the stable model solutions changes as system parameters are varied.

We considered seriously this comment but it is not that clear, for several reasons. First, bifurcation analysis usually applies to ODE system. We discuss rapidly such a simplified system, but this is not the heart of our work. Second, there is a lot of parameters: performing a bifurcation analysis would mean to select just one or two of them to play with. At this stage it is not clear how to select them.

Alternatively, a global sensitivity analysis revealing which parameters have the greatest effect on biologically relevant model outputs could be a useful addition to the work. Thus, the computational aspects do not seem to constitute a significant part of the paper's novelty.

We agree with this comment: the main novelty relies on the modelling discussion of the protumor effects, which is addressed through numerical experiments. Performing a global sensitivity analysis is definitely a valuable goal. But it requires first to set up relevant ranges for the parameters, which needs a preliminary calibration step, based on experimental data. This step in itself requires a complete analysis, with the development of dedicated optimization tools to handle the complex PDE system at hand. We have added concluding perspectives describing such directions.

For the reasons mentioned above, I do not consider the paper suitable for publication in PLOS Comp Biol. However, I would strongly encourage the authors to submit it elsewhere, eg J Theor Biol, where their previous work has been published. I include below some general comments which are intended to help the authors improve their manuscript.

General comments
• Introduction: The review of the literature is extremely limited. The seminal paper by Kuznetsov et al (1997) is not even cited. I would encourage the authors to include a broader review of the literature and explain how their work relates to (and extends) the existing literature. In particular, many other authors have developed (simpler) models which reproduce the 3Es of immuno-editing. They could also mention other modelling approaches, beyond continuum models (eg Agent-based approaches, stochastic models).

It is clearly not possible to be exhaustive and this paper is not written as a review on the subject. Based on this remark, we indeed discovered relevant papers by V. Kuznetsov that we are pleased to quote.

• Mathematical Model: Are the authors modelling tumor growth in vitro or in vivo?

Our model is intended to apply to in vivo tumor growth.

What is the source of the immune cells? Do pro- and anti-tumor immune cells come from the same source (this is explained later, on page 19)?

We have added a comment in the introduction to the model and make it precise that we can consider distinct sources of immune cells.

It would be helpful to include a schematic of the mathematical model. It would also be helpful to introduce the model geometry and domain from the outset and to explain why some dependent variables depend on time t and size z (and not spatial position x) while others depend on time t and spatial position x.

The revised version provides such figures containing the main elements of the modelling.

In particular, how can the authors justify neglecting spatial variation in the tumor cell density while chemokines and immune cell concentrations vary with x and t and the evolution on the tumor cell density at a particular spatial position depends on chemokine levels there?

A comment has been added to motivate the use of two length scales.

• Mathematical Model: the model development is difficult to understand. The authors start by introducing and defining a variety of functional forms (eg the Gompertz law, the cell division operator) before introducing the governing equations. Given the complexity of the model (which is quite intricate), I would suggest that the authors provide a brief description of their model in the main text (with supporting schematic) and relegate the details to an Appendix.

We have added an illustrative scheme but it seems difficult to us to relegate equations in the Appendix since the discussion refers a lot to the equations and their introduction: we definitely think that relevant information would be missing.

• Well-mixed model analysis: After presenting their model, the authors proceed to analyse a special case for which spatial effects are neglected and the governing equations reduce to a system of ODEs. A detailed (but standard) analysis of the steady state solutions of the reduced model is presented, together with linear stability analysis and some supporting numerical solutions of the ODE system. This is all fine but seems out of place in PLOS Comp Biology. Additionally (to improve the readability), I would suggest that the detailed analysis be relegated to Supplementary Material and merely summarise the key results in the main text. Similarly, Theorem 1 regarding existence of a unique steady state solution seems out of place in this journal.

We acknowledge that we probably miss appropriateness to the journal.

• Dimensionless parameter values are stated (Table 2) but no justification for the choices is provided.

• Figure 1: it would be helpful if the same scalings were use on the y-axis in panels (a), (b), (c) and d) – this would facilitate comparison of the results (same comment applies to subsequent figures).

We do not agree with this comment: since the values are very different, using the same y-scale would completely shrink the signal for a part of the parameters and the curves would not be readable at all.

• Results: the parameter sensitivity analyses are rather limited – the authors vary one parameter at a time and present time-courses showing how, eg, the tumour mass changes over time as particular parameters are varied. I would encourage the authors to consider a more detailed study of their model (eg bifurcation diagrams, global stability analysis).

We are absolutely convinced of these directions. Nevertheless, considering the complexity of the model and of the phenomena, such an objective is far beyond the aims of this paper and would require specific and very involved developments.

Reviewer #3: The authors addressed complex features of tumor-immune interactions by including both anti-tumor immune cells and pro-tumor immune cells. These are not simple interactions to model but it is critical that we do so. Considering the numerical effects of either enhancing anti-tumor immune cells or inhibiting pro-tumor immune cells is unique and unintuitive. Overall the paper is very well organized and clearly communicated. My expertise is immunology/biology. I will limit my comments as such and leave the deeper mathematical discussions for other reviewers.

A few considerations to improve the paper:
Model assumptions: T cells and natural killer cells are both lymphoid cells but NK cells are not antigen-specific.

This has been corrected.

The term "evolution" is used incorrectly here and throughout the paper. Evolution refers to selection of the most fit by selection pressures which requires births and deaths. This is very important in a paper that discusses the equilibrium phase of immunoediting as this is when the strongest selection for immune evasive phenotypes occurs.

Here "evolution" refers to the changes a quantity (a concentration, a mass) is subject to with time, due to several physical and biological mechanisms. It does not refer to Darwin-like mechanisms. These changes can be described by balance relations, that ultimately are translated into differential equations. When appropriate we have changed into 'dynamics'.

The term "antigenicity" is misused here.

It has been removed.

A.7 is an incorrect representation of biology. CD8 T cells are not recruited and activated by APCs. Naïve CD8 T cells enter the lymph nodes where they survey DCs. Activated DCs will increase the rate this happens but they do not recruit and this does not happen within tumor tissue. Please clarify. Why is recruitment/activation rate related to tumor mass? This is fine for the model but please explain the thinking behind this decision.

There has been a misunderstanding as we agree with the reviewer. We have rephrased A.7

It is unclear how the immune cells enter the tumor, through vasculature or migration through neighboring tissue. If from vessels then increased angiogenesis would increase supply of immune cells.

The proposed modeling does not enter into such details: the detailed geometry of the tumor is disregarded here. The supply of immune cells is mainly described through the directed motion induced by the chemotactic field. We have added a comment in this direction.

I am confused by the conversion of "some immune cells that eliminate tumor into pro-tumor immune cells" as it relates to Tregs.

As indicated it relates to TAM-M2 and TAN-N2.

Is the model measuring conversion of cytotoxic CD8 T cells converting into CD8+ Tregs (which are rare) or does it refer to helper CD4 T cells (which are not cytotoxic) being converted into induced Tregs (iTregs)? Please clarify.

We do not agree with these comments. The reviewer was misled.

Construction of the model – I agree that the tumor growth needs to be limited by size.

Sure. But in the earliest stages of tumor growth the limitation can be disregarded, with the advantage of not introducing additional (unknown) parameters in the model.

Page 8. Again the term evolution is misused, cytokines cannot evolve. Please be more precise in your wording.

See comment above.

Results:

Page 19. "This assumption describes the fact that protumor…" This statement is confusing, please clarify

With such an involved model the authors could have provided more information regarding the underlying dynamics and differential outcomes when treating to either rescue the exhausted anti-tumor immune cells or when inhibiting the pro-tumor immune cells.

Specifically - The authors highlight the effects of division rate on tumor control when critical mass is positive. But they do not explore the effects with the two treatment strategies. (m=0 is likely to occur in an inflamed environment, e.g. smokers lungs, or in a new metastatic lesion where the soil has been altered due to systemic effects caused by the primary)

Comments have been added in line with this observation.

Additionally, some tumors have very few exhausted cells to be rescued at the time of treatment and some immune cells do not stay in the exhausted compartment very long (they die or leave the tissue). The authors do not fully address how this is handled in the model.

This is handled by the parameters of the model, that can be tuned to describe such effects. Further comments have been added according to this remark.

The authors discuss and model the direct and indirect effects of pro-tumor immune cells on tumor growth yet they do not address if inhibiting one or the other would have differential effects on the system.

The main findings are that starting immunotherapy early works better than late. However, they do not state why this happens in the model… Larger tumor, more pro-tumor immune cells, poor effector to target ratio?

The paper is already quite long and indicate several issues for future works. Due to the huge number of parameters, choices should be made to isolate the most relevant questions, certainly with a better estimation of the parameters.

Conclusion
The conclusion could also benefit from some more input. The model includes many biological features yet the effects of these are not fully addressed. The paper would benefit from more interpretation and future vision by the authors.

Concluding perspectives have been added in the revised version.

Minor fixes "Natural Killer" should not be capitalized Labels on the figures are hard to read

Modified as suggested.
