# Supplementary material for: A size and space structured model of tumor growth describes a key role for protumor immune cells in breaking equilibrium states in tumorigenesis
Source: PLoS One. 2021 Nov 22;16(11):e0259291. doi: 10.1371/journal.pone.0259291 (PMC8608488; doi:10.1371/journal.pone.0259291)
Supplement: Attachment — Submitted filename: Report_PlosOne.pdf [file pone.0259291.s002.pdf]

Answer to the reviewers'reports on

A size and space structured model of tumor growth describes a key role for protumor immune cells in breaking equilibrium states in tumorigenesis

By Kevin Atsou, Fabienne Anjuère, Véronique M. Braud,Thierry Goudon

We thank the reviewers for their encouraging comments. We are pleased to take into account their recommendations. The main changes appear in blue in the revised version. In particular the captions of the figures all have been amplified. The revised version adopts the template of PlosOne. According to a recurrent reviewers observation, we have paid attention to make the structure of the paper clearer, and to make precise, in each section, which set of equations is under consideration.

Reviewer #1: The authors are interested in immunoediting in tumors and in particular in the escape phase corresponding to an imbalance between an anti-tumor response and a pro-tumor response. The dynamics of the tumor are entirely driven by the immune system. The phenomena of angiogenesis and interactions with other healthy cells are ignored. The proposed results are difficult to assess but appear biologically probable. This work makes it possible to understand the failure of certain immunotherapies. The fact that the solution to the cancer dynamic is based on the immune system does not allow us to understand the complexity of this pathology. It would have been interesting to combine this model with, for example, the process of angiogenesis and thus to test therapeutic combinations that already exist on the market.

Mathematical modeling can help in capturing the complexity of the tumor microenvironment (TME) and its role in driving tumor growth and dissemination. The success of cancer immunotherapies has highlighted the key role of the immune system. Mathematical modeling requires to simplify assumptions and we have focused here on the interaction of tumor and immune cells. We understand the frustration of the reviewer that other components are not explicitly included in the model but it is not realistic at this stage to include too many parameters. In the type of modeling adopted here, angiogenesis can be described very roughly, just by tuning some parameters, those linked to tumor cell growth and division and to protumor immune cells. Thus, discussing the effect of therapeutic combinations is highly relevant. An additional difficulty is to reach quantitative assessment as it relies on the calibration of parameters which are very poorly known. This step requires a specific work of investigation in itself. Such challenges will certainly be the object of future works.

Reviewer #2: The authors introduce a complex PDE model for anti-tumour/pro-tumour immune responses to cancer. Then, they consider a simplified version of this model (described by some simple ODEs) and for this model they investigate the existence and stability of various steady states. After that, the authors focus (I assume…) on the full model and try to find the equilibrium states. Then, the authors perform various numerical simulations showing what happens with model dynamics (although it is not clear whether they focus on the PDE or the ODE models) when various treatments are incorporated into the model.

Overall, the results have the potential to be very interesting. But the way they are presented makes it difficult to follow the flow of the paper. The authors should re-structure the paper and add more details, so that they "lead" the reader through their paper.

We acknowledge the difficulty of reading (and writing !) such an interdisciplinary article, that is expected to be of interest to several scientific communities. We made several trials of organization, considering seriously reviewer's recommendation, but did not find it easier to read, so we have kept the original organization. Going through the building of the mathematical modeling is required before we challenge the model with numerical simulations in the result section. We have revised the manuscript paying attention to clarify when PDE and ODE models are used, to make appear as neatly as possible the aims and scopes of each section and several sentences have been added to guide the reader throughout the article.

Below are some of the issues identified (which need to be addressed):

On page 11 the authors consider the case where "V and a are constant". How is this possible when on the previous pages the authors show that "V" and "a" depend on "z" (see equations (1), (2) and (3))?

There is no contradiction at all, a constant is just a specific case of a function of the variable z. Nevertheless, we fully agree that the original version could be a bit misleading. We have modified the paper in order to clarify the assumptions on the coefficients, and when we restrict to the constant case, which has the advantage, as indicated, to provide explicitly known equilibrium solutions for the PDE case. Concerning the discussion in p. 11 (original version), this assumption allows us to simplify the PDE model into an ODE system, which can be analyzed more easily. The ambition of this section is more clearly stated in the revised version. It is also indicated that the simplifying assumptions apply in this section only.

Page 14: I assume that the "healthy state (H)" is the one given by the zero vector on page 12? This needs to be made clear, since the notation (H) is never used on pages 12 and 13 ; only on page 14. Same comment about the other states: (NP) and (P).

The formula for the equilibria of the ODE system have now been labelled (H), (NP) and (N), with due reference within the text.

Figure 3: It is not clear what is with the "expected equilibrium value a/\delta (dotted)". The tumour mass seems to approach this value at t=50 (panel (a)), t=10 (panel(b)), t=2.5 (panel (c)) and t=1.5 (panel(d)). The authors need to show on these panels what happens for very large time. Cutting the figures exactly at the time point where the tumour mass approaches this dotted line does not explain what is going on in Figure 3. Please re-do figure 3 to show also tumour dynamics for large time "t".

It seems that the reviewer has been confused by the lack of captions and comments. We apologize for that. All captions have been modified accordingly. In fact, the equilibrium establishes *only* in fig. 3-(a): the concentration of immune cells shows damped oscillations around the expected equilibrium value. In the other cases, fig. 3(b), (c) and (d), we see the escape phenomena, with the blow up of the tumor mass while the concentration of immune cells does not reach the equilibrium value. The time scale changes because the blow up is very fast, the smaller a the faster the blow up. Figures with the same time scale would be simply

non-readable and with irrelevantly high values of tumor mass. We now provide detailed comments in the caption which are intended to clarify these issues.

The authors should also emphasise in the caption of this figure that there are two y-axes.

The captions have been revised and completed.

Finally, why is the continuous curve t-> mu1(t) but the dashed curve is t-> c (and not c(t))? Figure 4 (c)-(d): I cannot see the dotted line. I can see only a dashed line…Figure 4 (e)-(f): why the dashed curve is t->c while the continuous-dot curve is t->cr(t)? Why don't you have t->c(t)?

The figures and captions have been revised according to the comment.

Page 18: I don't understand how the new section "Existence of equilibrium phases" is connected to the previous section. Does the new section correspond to the full model? How are the results in this Section different from the results in [24], [35], … Please explain very clearly what you do here. The reader cannot be let to "guess" the results of the section/paper, and how they connect with each other.

We understand the possible confusion. We have added several comments to clarify the organization and which model is dealt with in all sections. The results in [35] considers the cell-division equation without any coupling: it analyses the eigenvalue problem. The results in [24] are restricted to a coupling with the antitumor cells. We address the extension of such a statement when adding protumor activities. This is also further commented in the revised version.

Figure 6: what represent the x-axis and y-axis in this figure? Same question for Figure 7.

The captions have been revised and completed.

Page 22: is "Emergent qualitative features …" a sub-section of the section "Results"? Or a parallel section? As discussed above, there is no explanation/flow for how the results connect with each other, so that the reader can follow easily this manuscript.

It is indeed unfortunate that the PlosOne template does not label sections and subsections. We have tried nevertheless to address this issue by adding announcements and comments intended to clarify the flow.

Figures 8,9,10, …14: Should I assume that these results are obtained with the PDE model, and the curves show space-averaged concentration values? Or do you show some simulations with an ODE model? Again, please don't expect the reader to "guess" what you do here. You need to explain in detail what you show in these Figures.

The captions have been revised and completed.

Can the authors show also some space-time snapshots corresponding to the most interesting behaviours seen in Figures 8-10? (if these figures actually show spatially-averaged cell concentrations …)

This point is indeed interesting and the revised version includes such figures showing the space organization of the tumor response, with dominant protumor activities next to the tumor in the situations when the control of the tumor is lost.

Figures 16, 17, …: Same question as before: do the curves show space-averaged concentrations of cells (i.e., tumour mass and immune response)?

Indeed the curves represent space-averaged quantities. The captions have been revised and completed to make the figures as unambiguous as possible.

Are the Sections on pages 31, 34, 36 actually sub-sections of the "Results" section? It would be easier if sections/sub-sections/sub-subsections would be labelled.

Absolutely: the section "Results" is made of several subsections. It is unfortunate that the PlosOne template does not label sections and subsections. We have added a paragraph, at the beginning of the Results section which announces the organization.
